# Coping Strategies among Patients with Dissociative Disorder: An Observational Study

**DOI:** 10.31729/jnma.8946

**Published:** 2025-04-30

**Authors:** Sulochana Joshi, Anup Raj Bhandari, Rabi Shakya

**Affiliations:** 1 Department of Psychiatry, Patan Academy of Health Sciences, Lagankhel, Lalitpur, Nepal

**Keywords:** *coping strategies*, *dissociative disorders*, *mental disorders: psychological stress*

## Abstract

**Introduction:**

Various factors contribute to the development of dissociative disorders. The ability to cope with different stressful events is key to symptom manifestations in this disorder. This study aims to explore various stressors and coping strategies in patients with dissociative disorder and the relationship between coping strategies with stressors and clinicodemographic characteristics.

**Methods:**

This was an observational cross-section study which evaluated patients with dissociative disorder presenting at the Department of Psychiatry in a tertiary care teaching hospital for 6 months (May to October 2017). We collected data on the demographic and clinical characteristics. We used the Presumptive Stressful Life Event Scale (PSLES) and Brief COPE scale to record the stressors and the coping responses, respectively. We summarized numerical variables with median and interquartile range (IQR) and categorical variables with proportions. Spearman rank correlation was run to determine the relationship between the PSLES and each coping strategy.

**Results:**

Of 108 patients, 86 (79.62%) patients were studied. 77 (89.53%) patients reported stressful life events, and failure in the examination was the most common stressor. Overall, coping strategies were used minimally. Problem-focused coping strategies were used slightly more frequently. There was no statistically significant correlation between stressors and coping strategies. Only religious coping was found to have a significant correlation with age.

**Conclusions:**

The majority had stressful events. The use of coping strategies was uncommon. Problem-focused coping strategies were used more frequently.

## INTRODUCTION

Dissociative disorder is a common condition with a lifetime prevalence of about 10%.^1^ It leads to significant socio-emotional and financial difficulties and loss of working days. Various factors contribute to the presentation of symptoms, including psychological stress, challenging situations, and the need for attention from others. In the Western population, childhood physical and sexual abuse have been commonly implicated.^2-5^ Additionally, vulnerability factors such as personality traits, illness behavior, and psychosocial functioning may also play a role. The ability to cope with different stressful events is key to symptom manifestation in dissociative disorders.

However, little is known about the coping strategies used by patients with dissociative disorders. In a cross sectional study exploring the perceived stress and coping strategies of Indian women with dissociative disorders, dysfunctional coping was significantly higher.^6^

This study aims to describe various stressors and coping strategies used by patients with dissociative disorders and to explore the relationship between coping strategies, stressors, and clinicodemographic characteristics. A better understanding of these strategies will aid in developing appropriate interventions and preventive measures.

## METHODS

This was an observational cross-section study carried out over six months (May 2017 to October 2017) in a tertiary care teaching hospital in Nepal. We conducted a post hoc power analysis to determine the statistical power of our correlation analysis, given the number of observations we included during the data collection period and the observed effect size we obtained during data analysis. We assessed whether our sample size was sufficient to detect the observed Spearman correlation coefficient at a two-tailed test with 95% significance level (a = 0.05). We used 10000 simulated datasets using Monte Carlo simulations to obtain an estimate of the statistical power. Ethical approval was obtained from the Institutional Review Committee (Date: 2017-04-17, Reference number: drs17004171122). Written informed consent was obtained from the patients (or immediate relatives as applicable). We assessed all the consecutive patients presenting to the in-patient and outpatient units of the Department of Psychiatry and the Emergency. Those diagnosed with dissociative disorder as per the International Classification of Diseases (ICD) 10 criteria were enrolled in the study. We interviewed the study participants using a semi-structured data collection tool and captured the data on demographics (age, gender, location, marital status, ethnicity, religion, occupation, and education) and clinical characteristics (presentation, diagnosis subtype, comorbidity, illness in the family, and stressors). We administered the Presumptive Stressful Life Events Scale (PSLES) to assign the scores to the stressful life events. PSLES is a tool, first described by colleagues from India, with 51 life events and their corresponding mean stress scores. ^7^ A clinical psychologist applied the Nepalese language-translated Brief Cope Scale in the second interview to record the responses on various coping strategies. Brief Cope Scale measures coping reactions which includes 28 items and 14 conceptually different coping styles.^8^

Data were entered in Microsoft Excel (Office 365, Microsoft Corporation, Washington, United States) and analyzed in R (R Core Team (2024). R: A language and environment for statistical computing. R Foundation for Statistical Computing, Vienna, Austria. The numerical variables were summarized using mean (and Standard Deviation) or median (and Inter-Quartile Range) as appropriate and the categorical variables were summarized with proportions. We categorized the 28 items of the Brief Cope Scale into 14 items and grouped the study patients into three categories - dysfunctional, emotional, and problem-solving.^9^ The PSLES and coping strategies were not normally distributed and therefore, a Spearman's correlation test was run to determine the relationship between the PSLES score and each of the 14 coping strategies, PSLES and age, and age and each of the 14 coping strategies.

## RESULTS

All the patients diagnosed with dissociative disorder during the six-month study period (May 2017 to October 2017) were screened; 108 patients were found eligible and were included in the study, of which 86 (79.62%) patients responded. Among the participants there were 9 (10.46%) male, 27 (37.50%) presented with unresponsiveness and 32 (37.21%) presented with mood disorder comorbidity.

**Table 1:** Demographic characteristics of study participants (n=86).

Characteristics	Percentage
**Gender**	
Male	9 (10.46)
Female	77 (89.53)
**Location**	
Rural	45 (52.32)
Urban	41 (47.67)
**Marital Status**	
Married	44 (51.16)
Single	42 (48.83)
**Ethnicity**	
Brahmin	38 (44.18)
Janjati	34 (39.53)
Chhetri	14 (16.27)
**Religion**	
Hindu	75 (87.2)
Christian	8 (9.30)
Buddhist	3 (3.48)
**Occupation**	
Semi-Skilled	38 (44.18)
Student	33 (38.37)
Business	7 (8.13)
Farming	4 (4.65)
Unemployed	2 (2.32)
Professional	2 (2.32)
**Education**	
Secondary School	49 (56.97)
Illiterate	11 (12.79)
Literate	11 (12.79)
Bachelors	8 (9.30)
Primary School	7 (8.13)

**Table 2:** Clinical characteristics of study participants (n=86).

Characteristics	n (%)
**Presentation**	
Unresponsiveness	27 (37.50)
Multiple physical symptoms	12 (16.66)
Abnormal behaviour	10 (13.88)
Mood problems	8 (11.11)
Unable to move	4 (5.55)
Abnormal movement of body	4 (5.55)
Pain	5 (6.94)
Forgetfulness	2 (2.77)
**Comorbidity**	
Psychiatric	50 (58.13)
No comorbidity	16 (18.60)
Both medical & Psychiatric	11 (12.79)
Medical	9 (10.46)
**Dissociative subtypes**	
Convulsions	40 (48.78)
Trans and possessions	18 (21.95)
Motor	10 (12.19)
Stupor	8 (9.75)
Amnesia	2 (2.43)
Amnesia and convulsion	2 (2.43)
Amnesia and stupor	1 (1.21)
Convulsion and stupor	1 (1.21)
**Dissociative subtype frequency**	
One	78 (95.12)
Two	4 (4.87)
**Illness in the family**	
Absent	62 (72.09)
Psychiatric	13 (15.15)
Similar	5 (5.81)
Medical	4 (4.65)
Both	2 (2.32)
**Number of stressors**	
Absent	14 (16.27)
One stressor	20 (23.25)
Two stressors	31 (36.04)
Three stressors	18 (20.93)
Four stressors	3 (3.48)

**Table 3:** Comorbid Psychiatric Condition in Study Participants (n=50).

Psychiatric Comorbidity	n (%)
Mood disorder	32 (37.21)
No Psychiatric Comorbidity	25 (29.07)
Stress related disorder	10 (11.63)
**Anxiety disorder**	9 (10.47)
Mood disorder and DSH	3 (3.49)
Psychotic disorder	2 (2.33)
**Stress related disorder and substance use**	2 (2.33)
Substance use disorder	2 (2.33)
Stress related disorder and DSH	1 (1.16)

DSH - Delibrate self-harm

**Figure 1:**
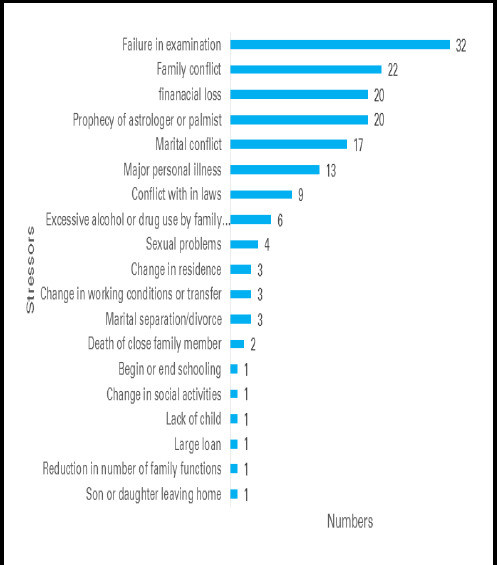
Distribution of Stressors in the study participants as per PSLES (n=50).

The median age was 23 (IQR: 16-32.25) years. The median PSLES score was 92.50 (IQR: 47-137). The highest score was 242. Stressful life events were reported by 77 (89.53%) patients and failure in the examination was the most common stressor (Figure 1).

Overall, coping strategies were used minimally by the patients, and a specific pattern could not be identified. Active coping 6 (IQR: 4-7), self-distraction 6 (IQR: 4-7), and emotional support 6 (IQR: 4-7) were among the coping strategies used. Similarly, humor 2 (IQR: 2-2), substance use 2 (IQR: 2-4), and behavioral disengagement 3 (IQR: 2-5) had lower usage.

Among the patterns of coping strategies, problem- focused coping included active coping 6 (IQR: 4-7), informational support 5 (IQR: 4-6), and planning 4 (IQR: 4-6). In emotion-focused coping, emotional support 6 (IQR: 4-7) and acceptance 5 (IQR: 3.25-6) were noted. Self-distraction 6 (IQR: 4-7) was among the dysfunctional coping strategies (Table 4).

**Table 4:** Central tendency measures of Coping strategies (n=50).

Pattern of Coping and Coping Strategy	Median (IQR)
**Dysfunctional Coping**	
Self Distraction	6 (4-7)
Denial	4 (3-6)
Substance Use	2 (2-4)
Behavioral Disengagement	3 (2-5)
Venting	4 (4-6)
Self Blaming	4 (2-6)
**Emotion-Focused Coping**	
Emotional Support	6 (4-7)
Positive Reframing	4 (3.25-6)
Humor	2 (2-2)
Acceptance	5 (3.25-6)
Religion	4 (3-6)
**Problem-Focused Coping**	
Active Coping	6 (4-7)
Informational Support	5 (4-6)
Planning	4 (4-6)

**Figure 2:**
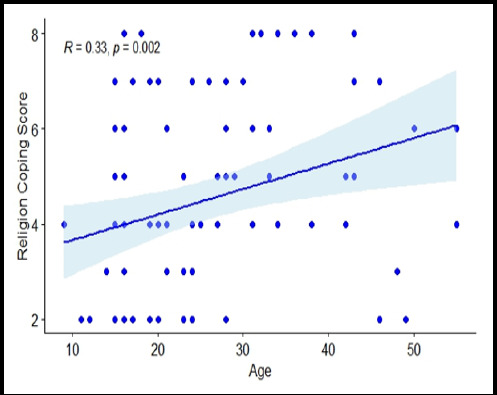
Spearman’s correlation between religious coping strategy and age

There was no statistically significant correlation between PSLES and each of the coping strategies. Only religious coping startegy was found to have a significant correlation with age, which was weak yet positive (ρ = 0.33, p = 0.002) (Figure 2). Using the Monte-Carlo simulations, we estimated a statistical power of 94%, which exceeds the customary 80% threshold typically considered adequate for detecting true effects. This indicates a high likelihood of identifying the observed correlation if a true relationship exists, signifying the robustness of our analysis.

## DISCUSSION

Our study aimed to explore the stressors and coping strategies of patients with dissociative disorders presenting in our center during the six-month study period. To the best of our knowledge, this is the first study on coping strategies among patients with dissociative disorders in the country. We found that the use of coping strategies was uncommon among patients with dissociative disorders. For those who used coping strategies, problem-focused coping strategies were used slightly more frequently. Any specific pattern of coping strategies used by the patients with dissociative disorders could not be identified.

Dissociative experiences have been found to increase with the increasing stress in the general population.^10,11^ Our study found a high rate of stressful life events compared to studies from India.^12-14^ This could be due to the inclusion of all different kinds of dissociative disorders of all ages. Perceived stress in women with dissociative disorder was high which is similar to our study.^6^ The mean score of PSLES in a study on dissociative convulsion in India was similar to the PSLES scores of our patients.^13^ Immediate stressful life events were reported preceding the dissociative symptoms by different studies similar to ours.^15-18^ Different kinds of stressors were revealed in our study with a maximum of 2 stressors in most of them. Failure in the exam was the most common stressor followed by family conflict, financial loss, and prophecy of an astrologer or palmist. Similar findings were reported from the study in Nepal with family conflict, broken family, and the death of a family member as the common stressors.^19^ Similar to our study interpersonal difficulties with family were important stressors in the study from Oman.^20^ A study from India reported family, study, and relationship issues that are similar to ours.^17^ Another Indian study reported marital and family conflict as well as death as the common stressors which have few similar stressors like ours.^14^ In contrast to our study, studies from the US, New Zealand, and Turkey showed childhood psychological trauma including childhood sexual, and physical abuse and neglect.^3, 21,^ ^22^ The reason for the abuses to be less in our study could be underreporting due to culture, unawareness, and stigma. Thus, our study showed that the stressors could be of various kinds and amounts in the patients with dissociative disorders given types of dissociative disorder, age, population, and culture.

Coping strategies on dissociative disorder are least studied. Our study showed that there is minimal use of coping strategies in addition to the use of various ineffective coping strategies by patients with dissociative disorders which is explained by the fact that the illness of dissociation hinders itself to exhibit coping strategy and also the patients with dissociative disorders have faulty coping mechanisms leading to symptoms of dissociation.^23^

Our study also showed the mixture of all three patterns of coping strategies in various proportions as well as suggestive of more problem-focused coping strategies used in dissociative disorders with comorbidities. This finding of our study contrasts with studies on coping strategies of dissociative disorders done in different places. A study done in India on women with dissociative disorders without comorbidities from 18 to 55 years showed dysfunctional coping strategies.^6^ A study from Pakistan on patients with all kinds of conversion disorder without comorbidities from 18 and above years showed the use of avoidance or emotion-focused and religious-focused coping.^24^ A study from US on dissociative convulsion without comorbidities from 18 and above years showed more religious, behavioral disengagement, and emotional venting coping strategies. A study from India on dissociative convulsion without comorbidities from 15 to 45 years from India showed more of self-controlling, distancing, escape avoidance coping strategies. Both of these studies showed a mixture of dysfunctional and emotion-focused coping strategies.^14,25^ A study from US dissociative convulsion without comorbidities showed more use of emotion-focused followed by dysfunctional than problem-focused coping strategies.^26^ A study from UK on dissociative convulsions with comorbidities showed seeking support, accepting responsibility, planful problem solving, and self-controlling as coping strategies.^23^ Our study differs from all the studies as our study had slightly more use of problem-focused coping strategies. The reason could be the study population, presence of comorbidities, including only one subtype of dissociative disorders in those studies, place of study, and scales used for the assessment of coping strategies. by patients with dissociative disorders.

Among all the coping strategies, only religious coping strategy was found to have a significant correlation with age. However, it was weak positive corelation. This could be explained by the semiskilled, Hindu female population in the study who could have taken into religiosity while dealing with difficult situations.

The reason for the less use of religious coping could be the overrepresentation of students and employed patients who might not have used religious practices as coping. Religious coping has shown various effects on stressful conditions and mental health.^27^ Our study also showed a positive correlation between PSLES and age. Similar to our study positive correlation was seen between age and stressors related to family and love affairs but a negative association was seen between age and study-related stressors.^17^ There was no significant correlation between PSLES and the coping strategies in our patients with dissociative disorder. Our finding was contrary to that of a study by Testa et al, which showed greater use of denial with greater distress.^25^ The reason for this difference could be that we had included all the subtypes of dissociative disorder where whereas the study by Testa et al had included only the dissociative convulsion subtype and an older population than ours. Thus our study showed that age played a role in dealing with the stressors and used religion to cope with stressors.

The use of a research tool that has not been formally validated is an important limitation of our study. While we made our best efforts to ensure its content validity, the absence of established reliability and validity metrics may introduce measurement bias. The generalization of the study is limited due to the sample size and setting of the study. However,the study is one of its kind in the dissociative disorder which adds to the literature on the coping strategies of the patients among dissociative disorders which is limited. Also the contrasting findings add broader view on the coping strategies on patieints with dissociative disorder, focussing on multiple coping strategies and not always dysfuncational and or emotion focussed coping strategies. Also highlights the need of careful interpretation of sociodemographic factors, stressors, comorbidities and dissociative phenomonen while linking the dissociative disorder.

## CONCLUSIONS

Coping strategies were used minimally among patients with dissociative disorders, with problem- focused strategies being slightly more common. Although there was a weak positive correlation between religious coping and age, no significant correlation was found between stressors and coping strategies. Given the exploratory nature of the study, further research is needed to better understand the relationship between comorbidities, coping strategies, and dissociative phenomena. Additionally, studies should explore coping strategies in the context of cultural, sociodemographic factors, and dissociative presentations.
